# Supplementary material for: BHLHE40/41 regulate microglia and peripheral macrophage responses associated with Alzheimer’s disease and other disorders of lipid-rich tissues
Source: Nat Commun. 2024 Mar 6;15:2058. doi: 10.1038/s41467-024-46315-7 (PMC10917780; doi:10.1038/s41467-024-46315-7)
Supplement: Supplementary file 1 — Supplementary Information [file 41467_2024_46315_MOESM1_ESM.pdf]

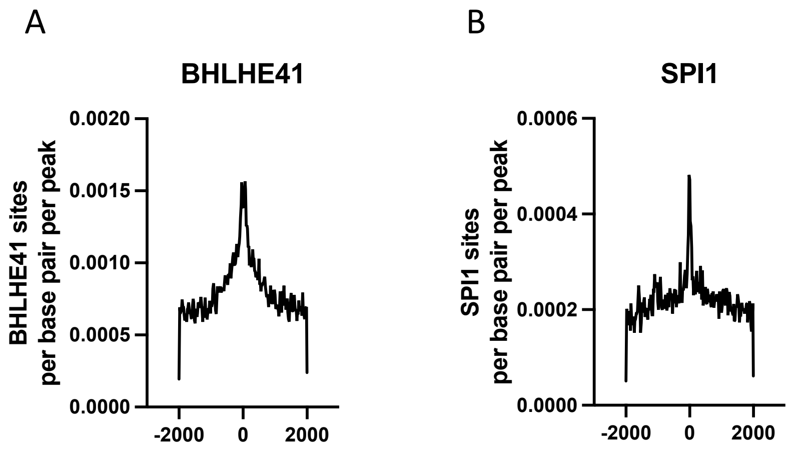

Supplementary Figure 1. BHLHE41 and SPI1/PU.1 regulons are most highly enriched in direct targets. Frequency of **A)** BHLHE41, **B)** SPI1/PU.1, motifs are plotted against the distance from the microglial ATAC-seq peaks[1] in the promoters of genes in their regulons.

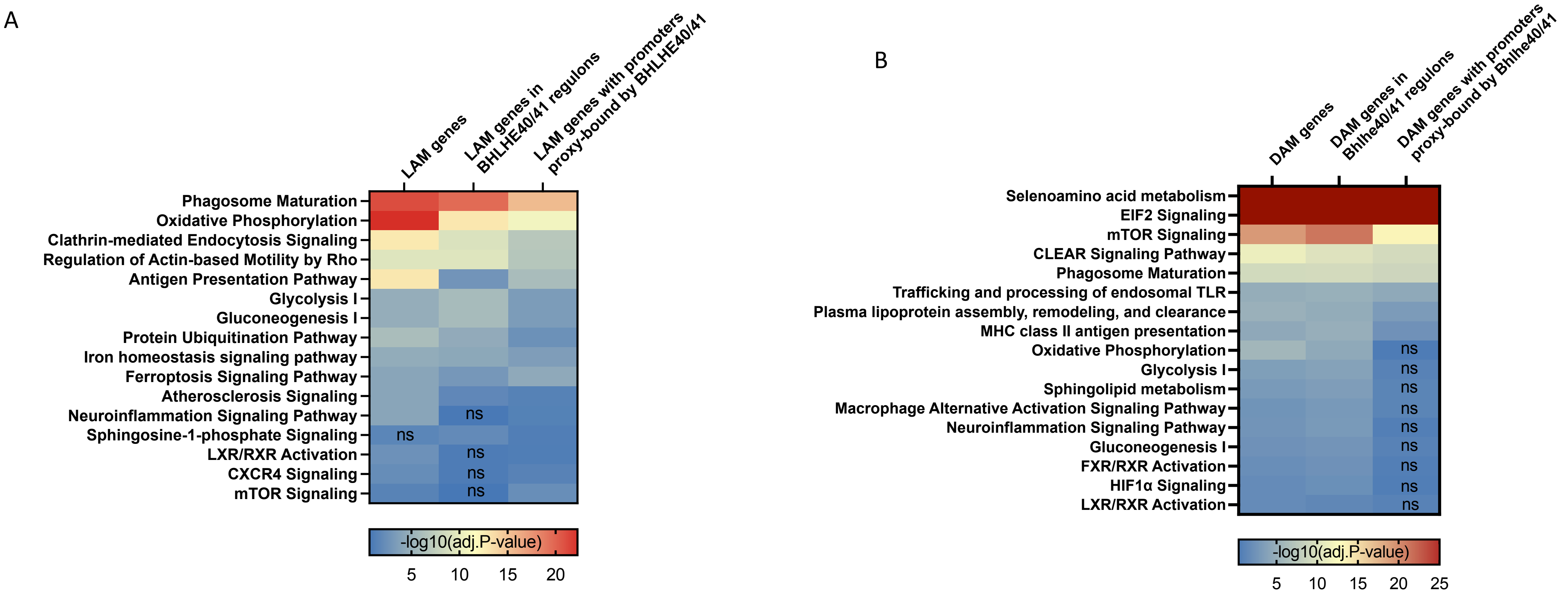

Supplementary Figure 2. Pathways shared between DLAM genes with promoter proxy-bound by BHLHE40/41 and DLAM genes in BHLHE40/41 regulons. Canonical pathways (IPA) shared between **A)** human LAM genes (Jaitin *et al*. [2], Dataset S6, FDR Adj.P-value < 0.05), LAM genes proxy-bound by BHLHE40/41, and LAM genes from BHLHE40/41 regulons. Adj.P-value represents Bonferroni-Holm correction. **B)** mouse DAM genes (Keren-Shaul *et al.,* [3], TableS2), DAM genes proxy-bound by Bhlhe40/41, and DAM genes from Bhlhe40/41 regulons. ns – non-significant (Adj.P-value > 0.05).

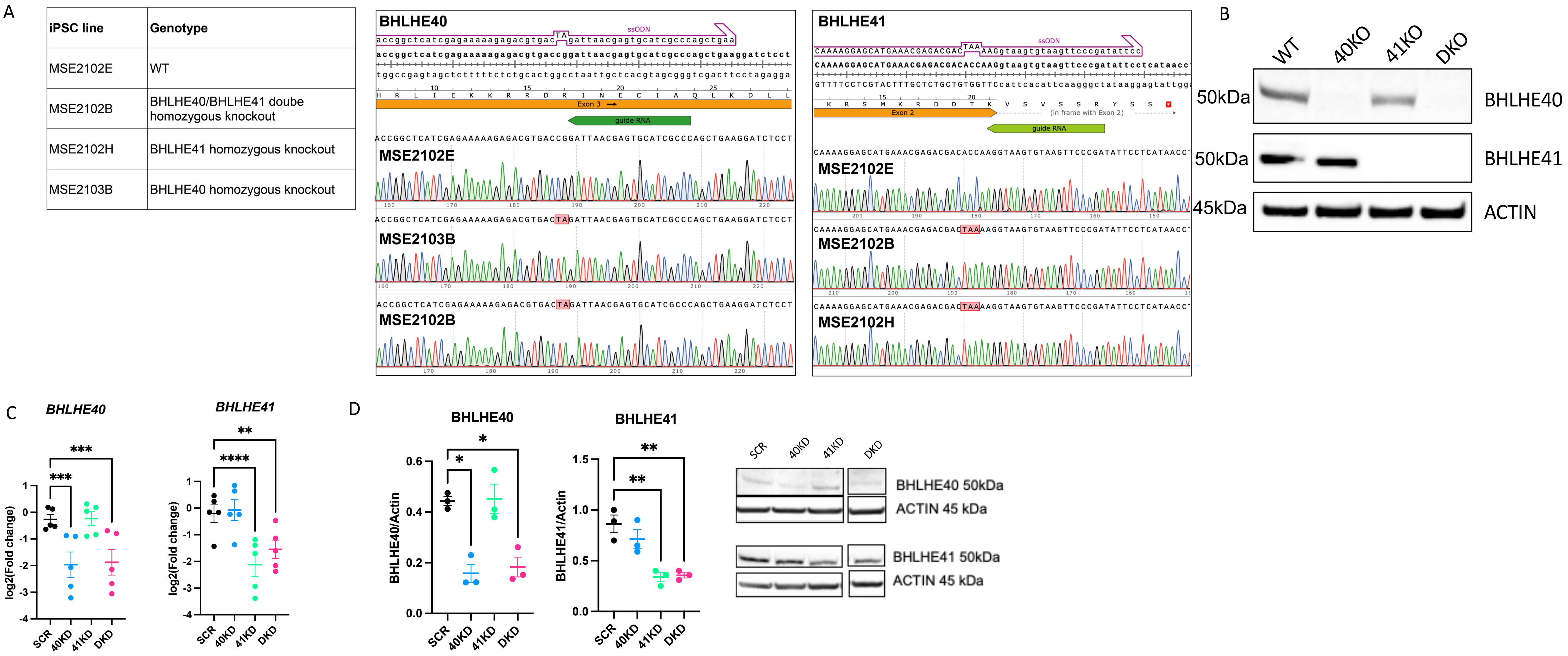

iPSC line	Genotype
MSE2102E	WT
MSE2102B	BHLHE40/BHLHE41 doube homozygous knockout
MSE2102H	BHLHE41 homozygous knockout
MSE2103B	BHLHE40 homozygous knockout

Supplementary Figure 3. Validation of knockout (KO) efficiency in human iPSC-derived microglia (iMGLs) with genetic inactivation of BHLHE40 and/or BHLHE41 and knockdown (KD) efficiency in human THP-1 macrophages (MACs) treated with BHLHE40 and/or BHLHE41 siRNAs. A) CRISPR/Cas9-mediated genome editing strategy to obtain homozygous BHLHE40 and/or BHLHE41 knockout human iPSC lines (Methods). **B)** Western blot confirming loss of BHLHE40 in 40KO and DKO iMGLs and loss of BHLHE41 in 41KO and DKO iMGLs. **C)** Expression of BHLHE40 and BHLHE41 measured by RT-qPCR, N=5/group. log2(fold change) (log2FC) is calculated with SCR MACs as reference. **D)** BHLHE40 and BHLHE41 normalized to Actin measured by western blot, N=3/group. Differences of means between groups were tested using one-way ANOVA with repeated measures followed by Dunnett's post-hoc test. * P-value < 0.05, ** P-value < 0.01, *** P-value < 0.001, **** P-value < 0.0001. Data plotted as mean ± SEM. Detailed statistics are shown in Supplementary File 1. 40KO = BHLHE40 KO iMGLs, 41KO = BHLHE41 KO iMGLs, DKO = BHLHE40 and BHLHE41 double KO iMGLs, WT = iMGLs derived from the parental iPSC line. 40KD = MACs treated with BHLHE40 siRNA, 41KD = MACs treated with BHLHE41 siRNA, DKD = MACs treated with BHLHE40 and BHLHE41 siRNA, SCR = MACs treated with scrambled siRNA.

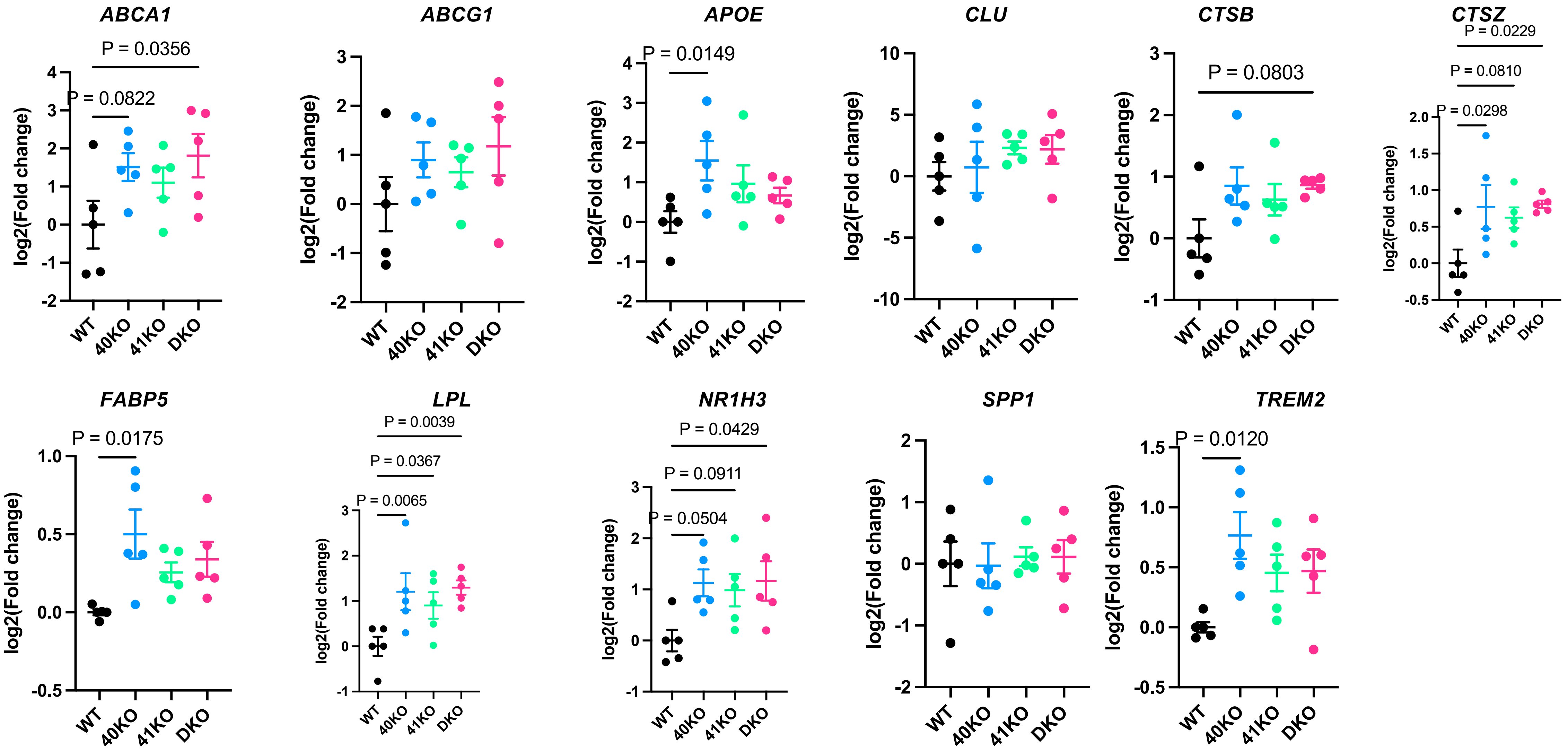

Supplementary Figure 4. Expression of lipid and lysosomal clearance genes in human iPSC-derived microglia (iMGLs) lacking *BHLHE40* and/or *BHLHE41*. Expression of lipid and lysosomal clearance genes measured by RT-qPCR, N=5/group. log2(fold change) (log2FC) is calculated with WT iMGLs as reference. Differences of means between groups were tested using one-way ANOVA with repeated measures followed by Dunnett's post-hoc test. * P-value < 0.05, ** P-value < 0.01. Data plotted as mean ± SEM. Detailed statistics are shown in Supplementary File 1. 40KO = BHLHE40 KO iMGLs, 41KO = BHLHE41 KO iMGLs, DKO = BHLHE40 and BHLHE41 double KO iMGLs, WT = iMGLs derived from the parental iPSC line.

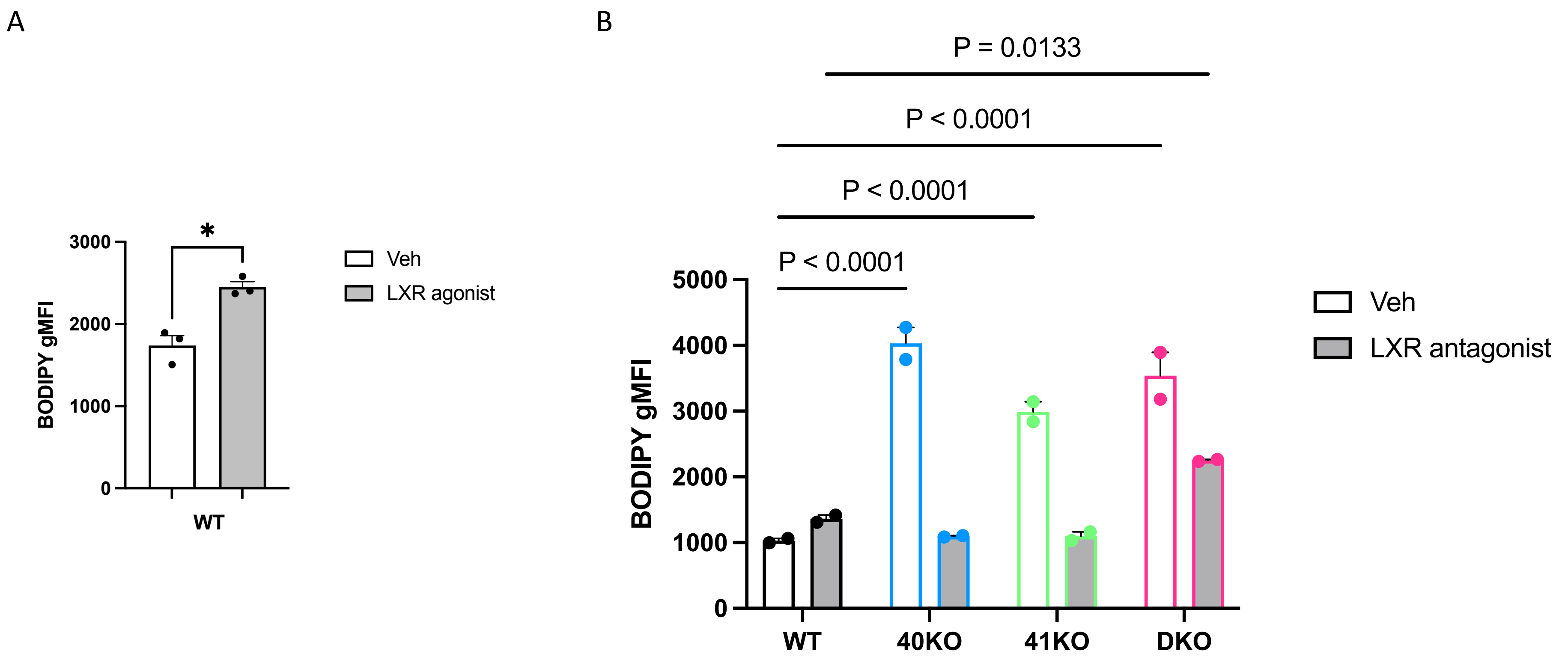

Supplementary Figure 5. LXR-dependent accumulation of lipid droplets (LDs) in human iPSC-derived microglia (iMGLs) lacking BHLHE40 and/or BHLHE41. A) LD content (BODIPY gMFI) measured by flow cytometry in BODIPY-positive WT iMGLs upon treatment with an LXR agonist (TO901317, 10uM, 48h) compared to vehicle control (Veh, DMSO), N=3/group. Differences of means between groups were tested using the paired t.test. * P-value < 0.05. **B)** LD content (BODIPY gMFI) measured by flow cytometry in BODIPY-positive WT, 40KO, 41KO, and DKO iMGLs treated with an LXR antagonist (GSK2033, 2uM, 24h) compared to vehicle control (Veh, DMSO), N=2/group. Differences of means between groups were tested using two-way ANOVA followed by Dunnett's post-hoc tests. *P-value<0.05, ****P-value<0.0001, ns – non-significant (P-value > 0.05). Data plotted as mean ± SEM. Detailed statistics are shown in Supplementary File 1. 40KO = BHLHE40 KO iMGLs, 41KO = BHLHE41 KO iMGLs, DKO = BHLHE40 and BHLHE41 double KO iMGLs, WT = iMGLs derived from the parental iPSC line.

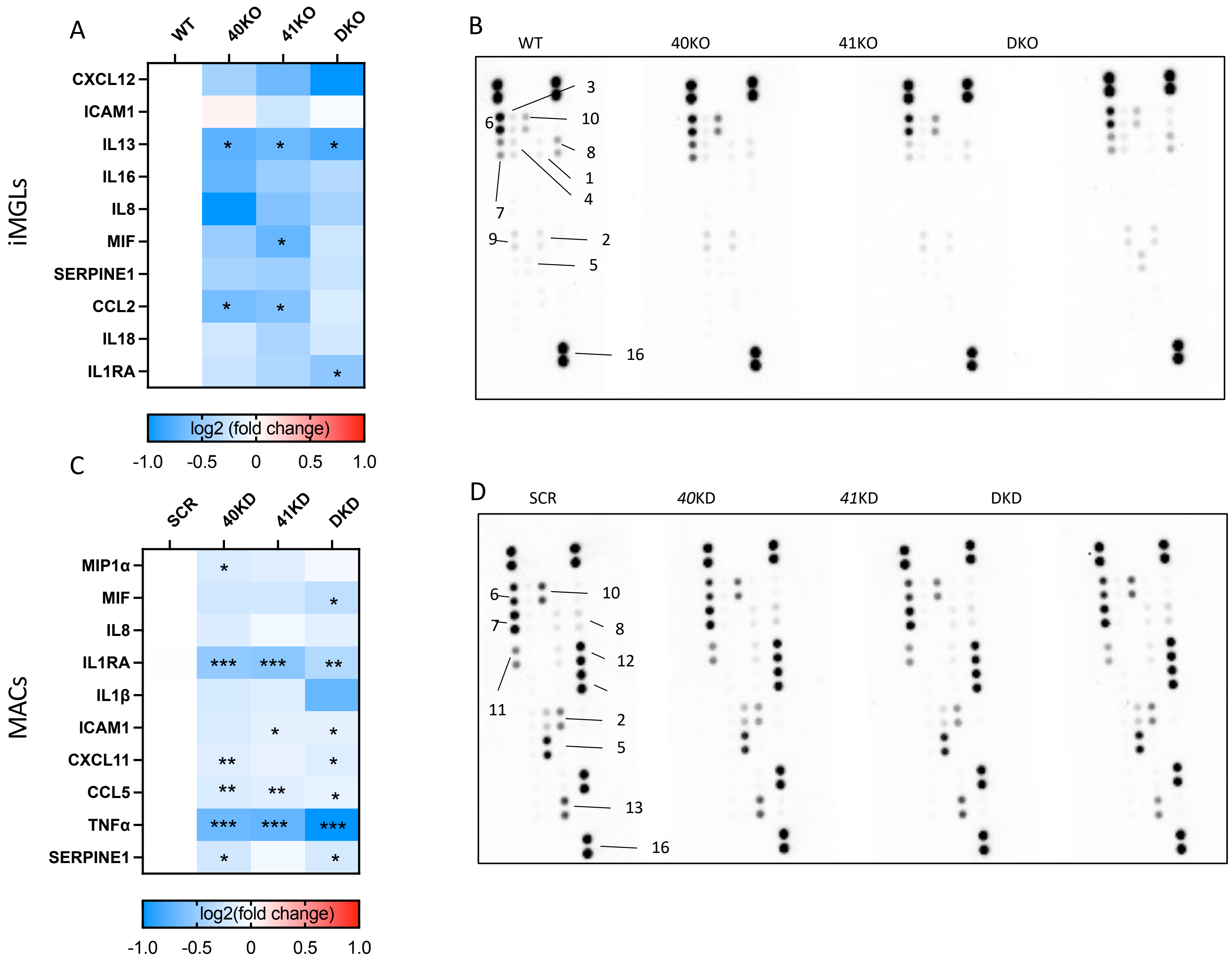

E

No.	Cytokine
1	CXCL12
2	ICAM1
3	IL13
4	IL16
5	IL8
6	MIF
7	SERPIN E1
8	CCL2
9	IL18
10	IL1RA
11	TNFα
12	MIP1α
13	IL1β
14	CCL5
15	CXCL11
16	Reference (ref)

Supplementary Figure 6. Reduce secretion of proinflammatory cytokines associated with complete loss (KO) or reduced levels (KD) of BHLHE40 and/or BHLHE41 in human iPSC-derived microglia (iMGLs) and THP-1 macrophages (MACs). Quantifications and dot blots of cytokines secreted in conditioned media from (A and B) human iPSC-derived microglia (iMGLs) with genetic inactivation of BHLHE40 and/or BHLHE41 or (C and D) human THP-1 macrophages (MACs) treated with BHLHE40 and/or BHLHE41 siRNAs measured using the Proteome Profiler Human Cytokine Array Kit (R&D Systems). **A and C)** Levels of cytokines secreted in conditioned media quantified as target density (i.e. mean target spot intensity multiplied by target spot area) normalized to reference density (i.e. mean reference spot intensity multiplied by reference spot area). log2(fold change) is calculated with (A) WT iMGLs or (C) SCR MACs as reference, N=3/group. **B and D)** Representative dot blots. **E)** Table of measured cytokines with corresponding dot identification number. Differences of means between groups were tested using one-way ANOVA with repeated measures followed by Dunnett's post-hoc test. *P-value<0.05, **P-value<0.01, ***P-value<0.001, ****P-value< 0.0001. Detailed statistics are shown in Supplementary File 1. 40KO = BHLHE40 KO iMGLs, 41KO = BHLHE41 KO iMGLs, DKO = BHLHE40 and BHLHE41 double KO iMGLs, WT = iMGLs derived from the parental iPSC line. 40KD = MACs treated with BHLHE40 siRNA, 41KD = MACs treated with BHLHE41 siRNA, DKD = MACs treated with BHLHE40 and BHLHE41 siRNA, SCR = MACs treated with scrambled siRNA.

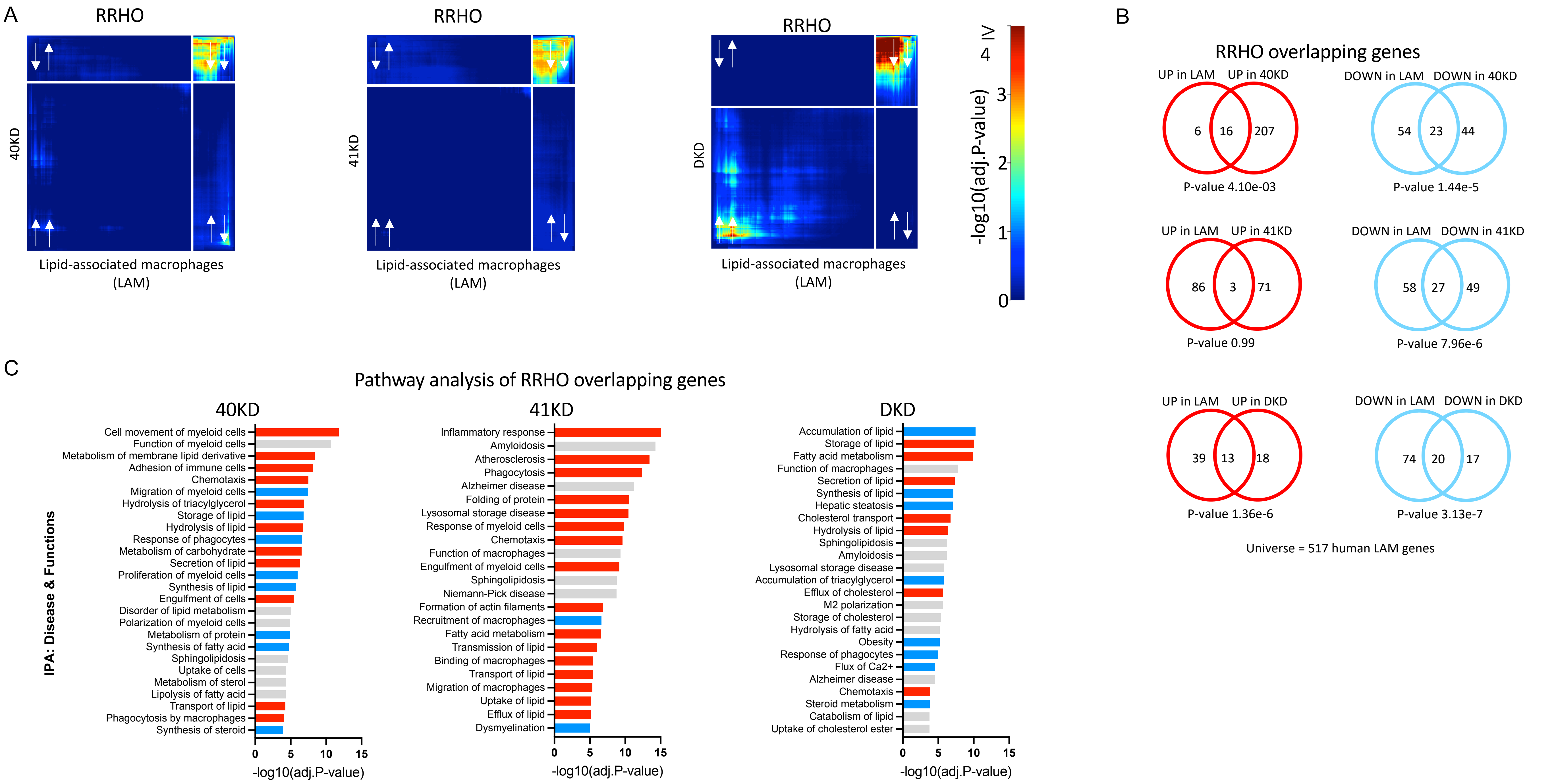

Supplementary Figure 7. Knockdown of BHLHE40/41 partially recapitulates LAM transcriptional and cellular responses in human THP-1 macrophages. A) Rank-rank hypergeometric overlap (RRHO) heatmaps visualizing significant overlaps in gene expression changes between each pair of BHLHE40/41 knockdown (KD) MAC and human LAM (Jaitin et al. [2], Dataset S6, FDR Adj.P-value < 0.05) transcriptional signatures. Adj.P-value in color temperature scale represents Benjamini-Hochberg corrected P-value of hypergeometric overlap test. White arrows indicate whether genes are up- or downregulated **B)** Venn diagrams of most significant overlaps between genes up-regulated in both *BHLHE40/41* KD MAC and human LAM transcriptional signatures, corresponding to the warmest pixel in the bottom-left quadrant of the respective heatmap (left) and Venn diagrams of most significant overlaps between genes down-regulated in both BHLHE40/41 KD MAC and human LAM transcriptional signatures, corresponding to the warmest pixel in the upper-right quadrant of the respective heatmap (right). P-values are calculated using the hypergeometric overlap test restricted to the universe of 517 human LAM genes (Jaitin *et al.* [2], Dataset S6, FDR Adj.P-value < 0.05). **C)** Pathways ("diseases and biological functions" category) found by IPA to be significantly enriched for RRHO overlapping genes. Red bars represent pathways predicted by IPA to be more active (i.e., with positive Z-scores), blue bars represent pathways predicted by IPA to be less active (i.e., with negative Z-scores), grey bars represent pathways with non-attributed Z-scores. Adj.P-value on the x-axis represents Bonferroni-Holm corrected P-value of pathway enrichment test. 40KD = MACs treated with BHLHE40 siRNA, 41KD = MACs treated with BHLHE41 siRNA, DKD = MACs treated with BHLHE40 and BHLHE41 siRNA, SCR = MACs treated with scrambled siRNA.

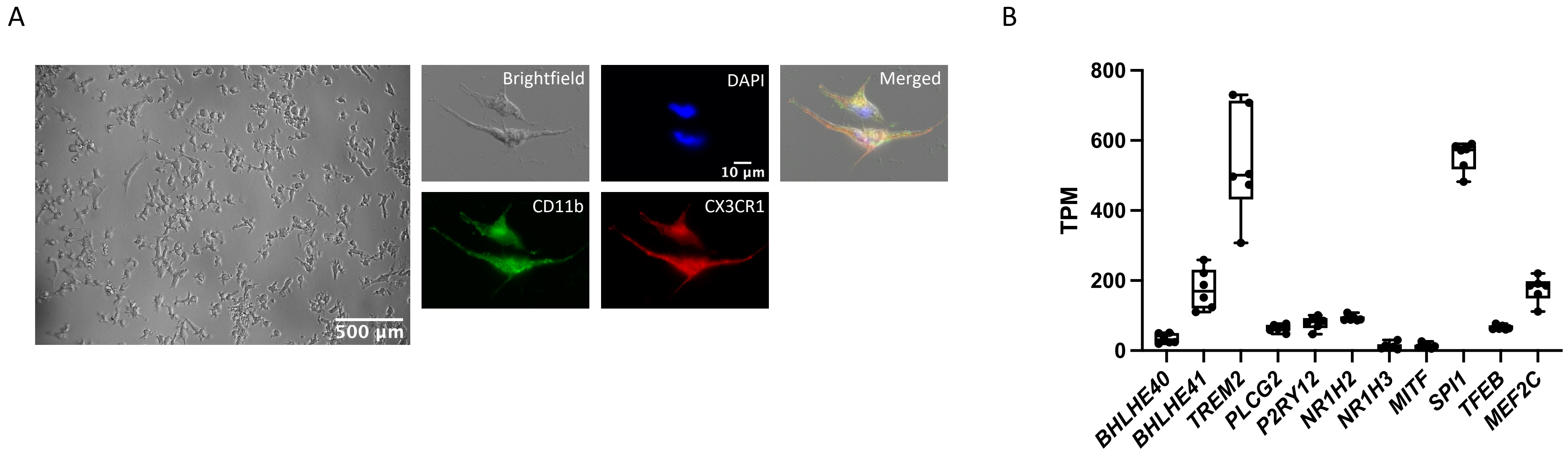

Supplementary Figure 8. **Differentiation of human iPSC lines into microglia-like cells (iMGLs). A)** Representative bright-field microscopic images and immunofluorescence staining for nuclei (DAPI) and differentiation markers (CD11b and CX3CR1) of a typical culture of mature iMGLs after 25d *in vitro*. **B)** Expression of *BHLHE40* and *BHLHE41* and other microglial genes (*P2RY12*, *PLCG2*, and *TREM2*) and transcription factors (*SPI1*, *MITF*, *MEF2C*, *NR1H2*, *NR1H3*, and *TFEB*) of interest measured by RNA-seq in iMGLs derived from the parental iPSC line, N=5/group. TPM = transcripts per million.

A

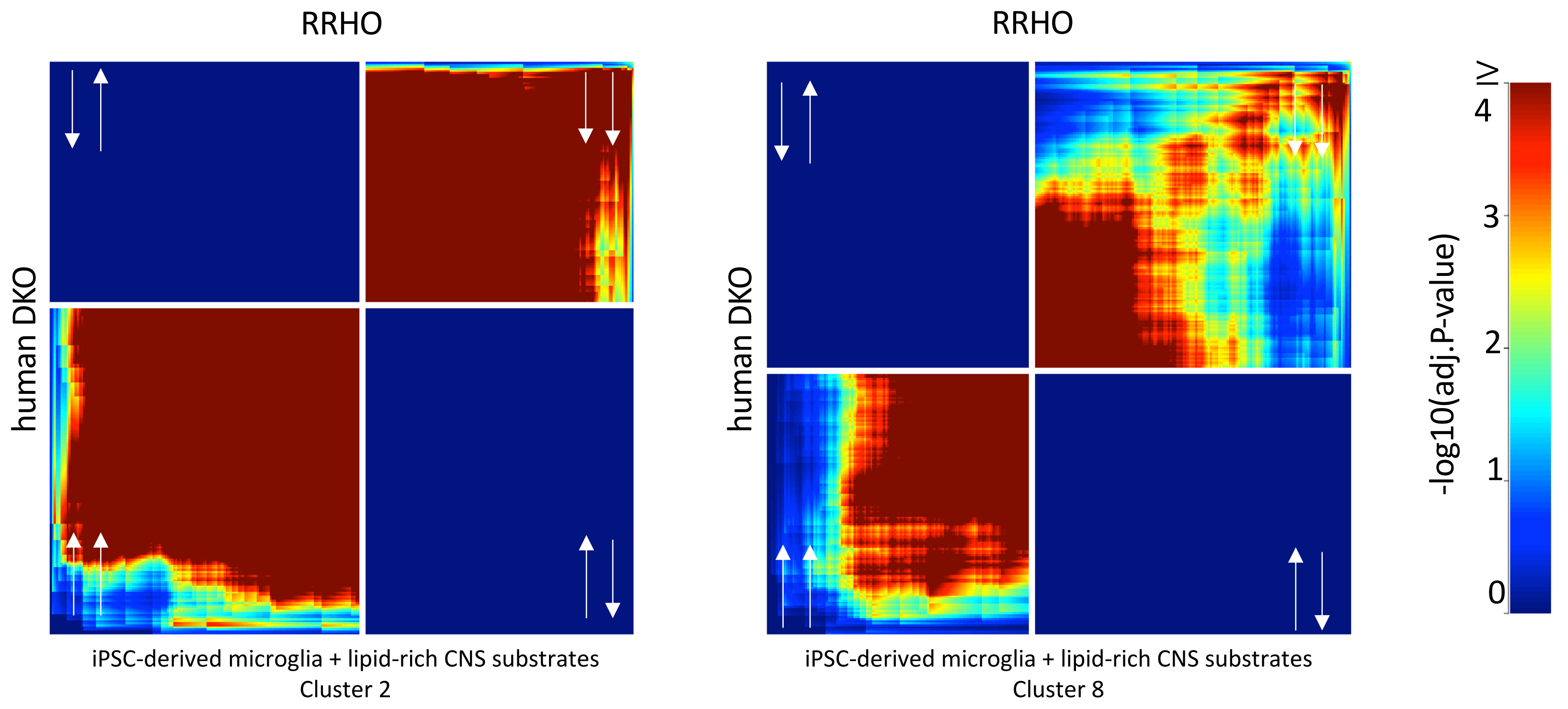

B

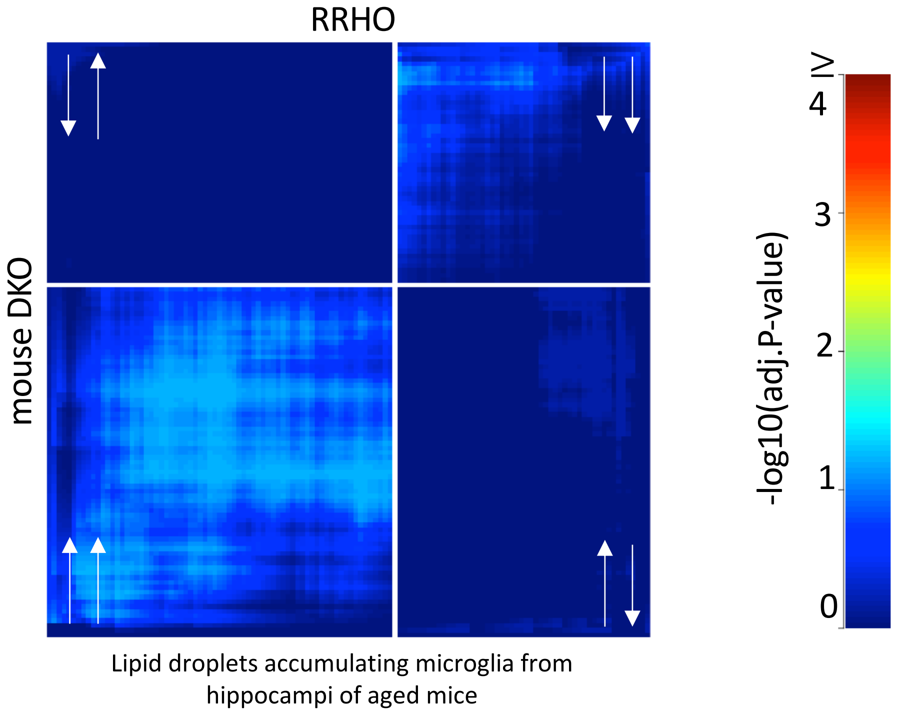

Supplementary Figure 9. Comparison of other data sets with transcriptional signatures of human and mouse microglial BHLHE40/41 knockouts. Rank-rank hypergeometric overlap (RRHO) heatmaps visualizing significant overlaps in gene expression changes between **A)** human BHLHE40/41 double knockout (human DKO) and human iPSC-derived microglia exposed to lipid-rich CNS substrates (Dolan et al. [4] Supplementary Table 2, Cluster 2 and Cluster 8) transcriptional signatures **B)** mouse Bhlhe40/41 double knockout (mouse DKO) and mouse lipid droplets accumulating microglia from hippocampi of aged mice [5] Supplementary Table T2-1). Adj.P-value in color temperature scale represents Benjamini-Hochberg corrected P-value of hypergeometric overlap test.

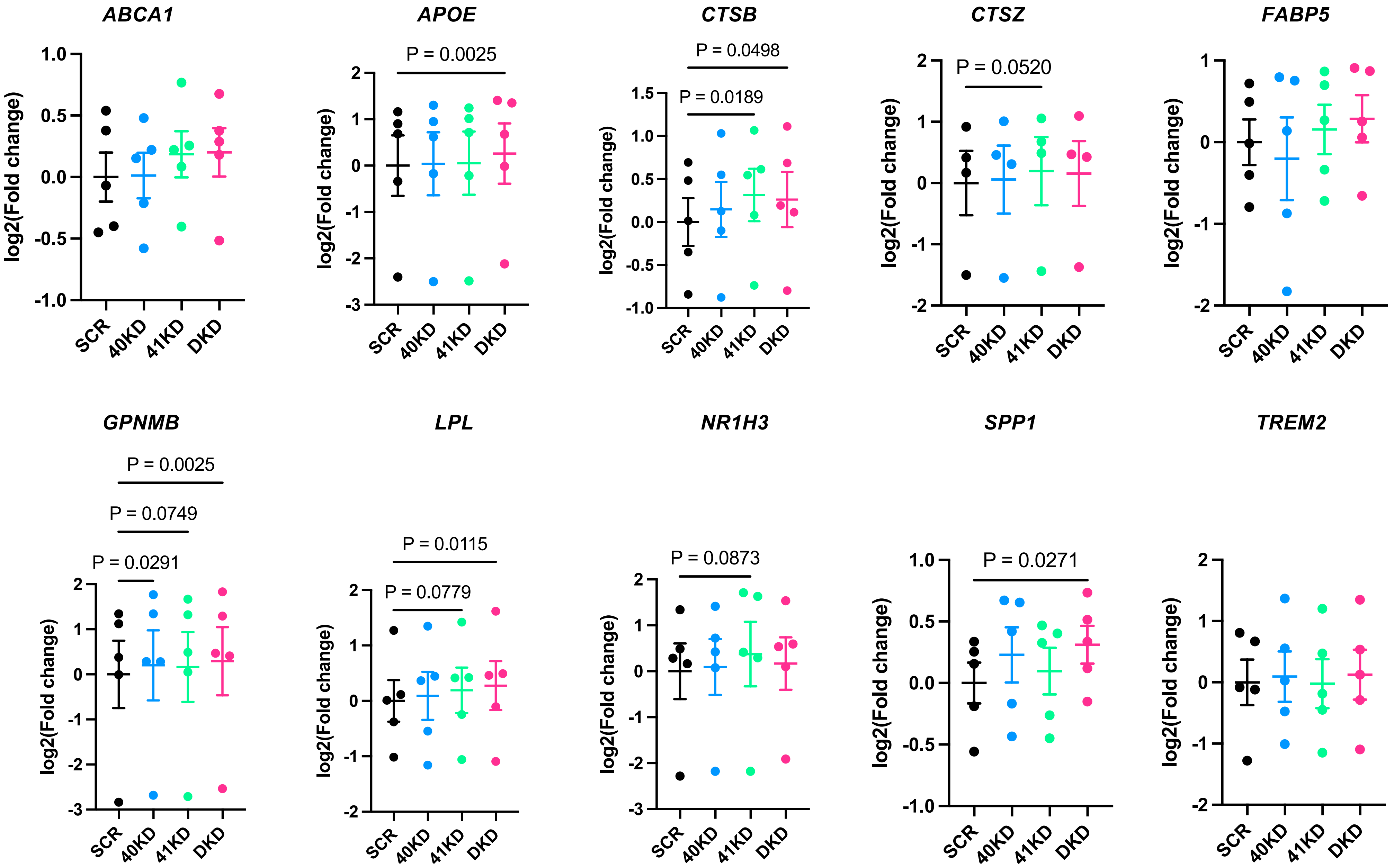

Supplementary Figure 10. Expression of lipid and lysosomal clearance genes in human THP1 macrophages (MACs) transiently transfected with siRNA targeting *BHLHE40* and/or *BHLHE41*. Expression of lipid and lysosomal clearance genes measured by RT-qPCR, N=5/group. log2(fold change) (log2FC) is calculated with SCR MACs as reference. Differences of means between groups were tested using one-way ANOVA with repeated measures followed by Dunnett's post-hoc test. * P-value < 0.05, ** P-value < 0.01. Data plotted as mean ± SEM. 40KD = BHLHE40 KD MACs, 41KD = BHLHE41 KD MACs, DKD = BHLHE40 and BHLHE41 double KD MACs, SCR = scrambled MACs

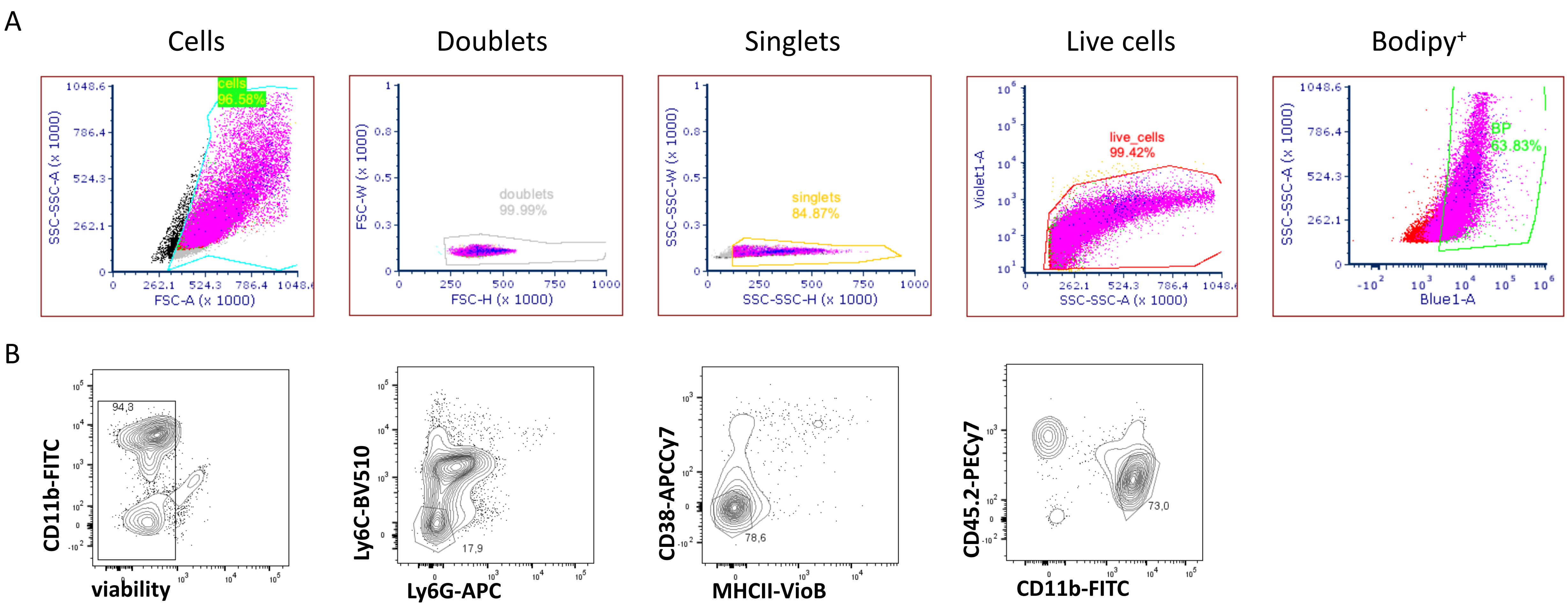

Supplementary Figure 11. Gating strategy for experiments listed in Figure 5, Figure 6 and Figure 7 (prior to RNAseq). A) Gating strategy to obtain BODIPY positive cells in iPSC-derived microglia (iMGLs) and THP-1 macrophages; similar strategy was used to gate LysoTracker, LysoSensor and DQ-BSA positive cells. **B)** Gating strategy to sort acutely isolated microglia from mouse brains for bulk RNAseq. Antibodies are listed in Methods and reporting summary.

References used in Supplementary Figures:

1. Gosselin, D. et al. An environment-dependent transcriptional network specifies human microglia identity. 3222, (2017). (ref.31 in the main text)
2. Jaitin, D. A. et al. Lipid-Associated Macrophages Control Metabolic Homeostasis in a Trem2-Dependent Manner. Cell 178, 686–698.e14 (2019). (ref. 6 in the main text)
3. Keren-Shaul, H. et al. A Unique Microglia Type Associated with Restricting Development of Alzheimer’s Disease. Cell 169, 1276–1290.e17 (2017). (ref. 8 in the main text)
4. Dolan, M.-J. et al. Exposure of iPSC-derived human microglia to brain substrates enables the generation and manipulation of diverse transcriptional states in vitro. Nat. Immunol. 24, 1382–1390 (2023). (ref. 32 in the main text)
5. Marschallinger, J. et al. Lipid-droplet-accumulating microglia represent a dysfunctional and proinflammatory state in the aging brain. Nat. Neurosci. 23, 194–208 (2020). (ref. 60 in the main text)

Supplementary File 1:

Figure legends with detailed statistical tests used in Figure 5, Figure 6, Supplementary Figure 3, Supplementary Figure 4, Supplementary Figure 5, Supplementary Figure 6

Figure 5. Knockout of BHLHE40/41 increases expression of lipid and lysosomal clearance genes, cholesterol efflux and lipid droplet content, lysosomal mass and degradative capacity in human iPSC-derived microglia (iMGLs).

A. **Expression of lipid and lysosomal clearance genes measured by RT-qPCR.** from N=5 independent iMGL differentiations/group (separate plots and statistical details for each gene in Supplementary Figure 4 and in the legend for Supplementary Figure 4 below). Post-hoc tests * P-value < 0.05, ** P-value < 0.01
B. **Intracellular APOE normalized to Actin measured by western blot.** Differences of means between groups were tested with one-way repeated measures ANOVA F(3,6)=10.66, P-value=0.0081, N=3 independent iMGL differentiations/group, followed by Dunnett's post-hoc tests: $ES_{40KO-WT}$=1.04 [0.43, 1.68], P-$value_{40KO-WT}$=0.0050; $ES_{41KO-WT}$=0.46 [-0.48, 0.77], P-$value_{41KO-WT}$=0.8204; ES_{DKO-WT}=0.98 [-0.28, 0.97], P-$value_{DKO-WT}$=0.2906.
C. **ABCA1 normalized to Actin measured by western blot.** Differences of means between groups were tested with one-way repeated measures ANOVA F(3,6)=13.11, P-value=0.0048, N=3 independent iMGL differentiations/group, followed by Dunnett's post-hoc tests: $ES_{40KO-WT}$=0.17 [-0.04, 0.38], P-$value_{40KO-WT}$=0.1068; $ES_{41KO-WT}$=0.42 [0.21, 0.63], P-$value_{41KO-WT}$=0.0021; ES_{DKO-WT}=0.14 [-0.06, 0.35], P-$value_{DKO-WT}$=0.1701.
D. **Secreted APOE normalized to total protein measured by ELISA.** Differences of means between groups were tested with one-way repeated measures ANOVA F(3,9)=6.41, P-value=0.0130, N=5 independent iMGL differentiations/group, followed by Dunnett's post-hoc tests: $ES_{40KO-WT}$=5.76 [2.03, 9.49], P-$value_{40KO-}$

$_{WT}$=0.0048; $ES_{41KO-WT}$=3.53 [-0.19, 7.23], P-value$_{41KO-WT}$=0.0630; ES_{DKO-WT}=2.93 [-0.79, 6.66], P-value$_{DKO-WT}$=0.1271.

E. **Percentage of cholesterol efflux.** Differences of means between groups were tested with one-way repeated measures ANOVA F(3,12)=24.68, P-value<0.0001, N=6 independent iMGL differentiations/group, followed by Dunnett's post-hoc tests: $ES_{40KO-WT}$=6.80 [3.76, 9.84], P-value$_{40KO-WT}$=0.002; $ES_{41KO-WT}$=7.80 [4.76, 10.84], P-value$_{41KO-WT}$<0.0001; ES_{DKO-WT}=8.80 [5.76, 11.84], P-value$_{DKO-WT}$<0.0001.

F. **LD content (BODIPY gMFI) measured by flow cytometry in BODIPY-positive cells** (top). Differences of means between groups were tested with one-way repeated measures ANOVA F(3,15)=7.23, P-value=0.0032, N=6 independent iMGL differentiations/group, followed by Dunnett's post-hoc tests: $ES_{40KO-WT}$=1928 [783.7, 3072], P-value$_{40KO-WT}$=0.0014; $ES_{41KO-WT}$=1151 [7.441, 2295], P-value$_{41KO-WT}$=0.0484; ES_{DKO-WT}=1546 [401.8, 2690], P-value$_{DKO-WT}$=0.0082. Representative flow-cytometry histograms (bottom)

G. **Lysosomal mass (LysoTracker gMFI) measured by flow cytometry in LysoTracker-positive cells** (top). Differences of means between groups were tested with one-way repeated measures ANOVA F(3,12)=2.81, P-value=0.0845, N=5 independent iMGL differentiations/group, followed by Dunnett's post-hoc tests: $ES_{40KO-WT}$=1987 [-1982, 5957], P-value$_{40KO-WT}$=0.4270; $ES_{41KO-WT}$=3125 [-844.9, 7094], P-value$_{41KO-WT}$=0.1341; ES_{DKO-WT}=4080 [109.9, 8049], P-value$_{DKO-WT}$=0.0438. Representative flow-cytometry histograms (bottom)

H. **Lysosomal acidification (LysoSensor gMFI) measured by flow cytometry in LysoSensor-positive cells** (top). Differences of means between groups were tested with one-way repeated measures ANOVA F(3,12)=4.41, P-value=0.0261, N=5 independent iMGL differentiations/group, followed by Dunnett's post-hoc tests: $ES_{40KO-WT}$=1327 [-19426, 22080], P-value$_{40KO-WT}$=0.9963; $ES_{41KO-WT}$=2858 [-17895, 23611], P-value$_{41KO-WT}$=0.9661; ES_{DKO-WT}=24256 [3502, 45009], P-value$_{DKO-WT}$=0.0222. Representative flow-cytometry histograms (bottom)

I. **Lysosomal proteolysis (DQ-BSA gMFI) measured by flow cytometry in DQ-BSA-positive cells** (top). Differences of means between groups were tested with

one-way repeated measures ANOVA F(3,12)=9.48, P-value=0.0017, N=5 independent iMGL differentiations/group, followed by Dunnett's post-hoc tests: $ES_{40KO-WT}$=3846 [-961.2, 8653], P-$value_{40KO-WT}$=0.1265; $ES_{41KO-WT}$=3125 [5.313, 9620], P-$value_{41KO-WT}$=0.0497; ES_{DKO-WT}=9485 [4678, 14293], P-$value_{DKO-WT}$=0.0005. Representative flow-cytometry histograms (bottom).

J. **DQ-BSA red fluorescent signal (total integrated density) measured over time using the Incucyte S3 live imaging system.** Differences of means between groups were tested with two-way repeated measures ANOVA F(3.8)=13.93, P-value=0.0015, N=3 independent iMGL differentiation/group, followed by Dunnett's post-hoc tests: $ES_{40KO-WT}$=37.34 [-106.7, 181.4], P-$value_{40KO-WT}$=0.7997; $ES_{41KO-WT}$=192 [47.91, 336.0], P-$value_{41KO-WT}$=0.0126; ES_{DKO-WT}=281.6 [137.5, 425.6], P-$value_{DKO-WT}$=0.0013. Total integrated density was calculated as mean red fluorescent intensity multiplied by surface area of masked object (i.e. cell), [RCU x μm^2].

Effect sizes (ES) are reported as unstandardized point estimates with 95% confidence intervals in the same unit as depicted on each graph.

Figure 6. Knockdown of BHLHE40/41 increases expression of lipid and lysosomal clearance genes, cholesterol efflux and lipid droplet content in human THP1 macrophages (MACs).

A. **Expression of lipid and lysosomal clearance genes measured by RT-qPCR.** from N=7 independent MAC differentiation (transient transfection with siRNA)/group. Post-hoc tests * P-value < 0.05, ** P-value < 0.01
B. **Secreted APOE normalized to total protein measured by ELISA.** Differences of means between groups were tested with one-way repeated measures ANOVA F(3,15)=2.52, P-value=0.0976, N=6 independent MAC differentiations/transient transfection with siRNA per group, followed by Dunnett's post-hoc tests: $ES_{40KD-SCR}$=2.33 [0.04, 4.62], P-$value_{40KO-WT}$=0.0457; $ES_{41KD-SCR}$=0.66 [-1.62, 2.94], P-$value_{41KD-SCR}$=0.7914; $ES_{DKD-SCR}$=0.8324 [1.45, 3.12], P-$value_{DKD-SCR}$=0.6689.

C. **Percentage of cholesterol efflux.** Differences of means between groups were tested with one-way repeated measures ANOVA $F(3,15)=22.82$, P-value<0.0001, N=6 independent MAC differentiations/transient transfection with siRNA per group, followed by Dunnett's post-hoc tests: $ES_{40KD\text{-}SCR}=12.45$ [8.47, 16.44], $P\text{-value}_{40KO\text{-}WT}<0.0001$; $ES_{41KD\text{-}SCR}=4.83$ [0.84, 8.81], $P\text{-value}_{41KD\text{-}SCR}=0.0170$; $ES_{DKD\text{-}SCR}=6.906$ [2.92, 10.89], $P\text{-value}_{DKD\text{-}SCR}=0.0011$.

D. **LD content (BODIPY gMFI) measured by flow cytometry in BODIPY-positive cells.** Differences of means between groups were tested with one-way repeated measures ANOVA $F(3,15)=10.31$, P-value=0.0006, N=6 independent MAC differentiations/transient transfection with siRNA per group, followed by Dunnett's post-hoc tests: $ES_{40KD\text{-}SCR}=2348$ [567.0, 4128], $P\text{-value}_{40KO\text{-}WT}=0.0097$; $ES_{41KD\text{-}SCR}=2890$ [1110, 4671], $P\text{-value}_{41KD\text{-}SCR}=0.0020$; $ES_{DKD\text{-}SCR}=3563$ [1782, 5343], $P\text{-value}_{DKD\text{-}SCR}=0.0003$. Gates were drawn based on fluorescence minus one (FMO) controls.

Effect sizes (ES) are reported as unstandardized point estimates with 95% confidence intervals in the same unit as depicted on each graph.

Supplementary Figure 3. Validation of knockout (KO) efficiency in human iPSC-derived microglia (iMGLs) with genetic inactivation of BHLHE40 and/or BHLHE41 and knockdown (KD) efficiency in human THP-1 macrophages (MACs) treated with BHLHE40 and/or BHLHE41 siRNAs.

A. CRISPR-Cas9 genome editing strategy to obtain homozygous BHLHE40 and/or BHLHE41 knockout human iPSC lines.

B. Western blot confirming loss of BHLHE40 in 40KO and DKO iMGLs and loss of BHLHE41 in 41KO and DKO iMGLs.

C. **Expression of BHLHE40 and BHLHE41 measured by RT-qPCR, N=5/group. log2(fold change) (log2FC) is calculated with SCR MACs as reference.** Differences of means between groups were tested with one-way repeated measures followed by Dunnett's post-hoc tests

BHLHE40: ANOVA F(3,12)=20.02, P-value<0.0001, N=5 independent MAC differentiations/transient transfection with siRNA per group, followed by Dunnett's post-hoc tests: $ES_{40KD-SCR}$=-1.699 [-2.498, -0.8999], P-$value_{40KO-WT}$=0.0003; $ES_{41KD-SCR}$=0.0283 [-0.7707, 0.8274], P-$value_{41KD-SCR}$=0.9993; $ES_{DKD-SCR}$=-1.606 [-2.405, -0.8073], P-$value_{DKD-SCR}$=0.0005.

BHLHE41: ANOVA F(3,12)=25.21, P-value<0.0001, N=5 independent MAC differentiations/transient transfection with siRNA per group, followed by Dunnett's post-hoc tests: $ES_{40KD-SCR}$=0.1356 [-0.6250, 0.8963], P-$value_{40KO-WT}$=0.9318; $ES_{41KD-SCR}$=1.912 [-2.673, 1.151], P-$value_{41KD-SCR}$<0.0001; $ES_{DKD-SCR}$=-1.338 [-2.098, -0.5770,], P-$value_{DKD-SCR}$=0.0014.

D. **BHLHE40 and BHLHE41 normalized to Actin measured by western blot**

Differences of means between groups were tested with one-way repeated measures followed by Dunnett's post-hoc tests

BHLHE40*:* ANOVA F(3,6)=12.51, P-value<0.0054, N=3 independent MAC differentiations/transient transfection with siRNA per group, followed by Dunnett's post-hoc tests: $ES_{40KD-SCR}$=-0.2847 [-0.4832, 0.0862], P-$value_{40KO-WT}$=0.0107; $ES_{41KD-SCR}$=-0.0091 [-0.1894, 0.2076], P-$value_{41KD-SCR}$=0.9977; $ES_{DKD-SCR}$=-0.2599 [-0.4584, -0.0613], P-$value_{DKD-SCR}$=0.0162.

BHLHE41*:* ANOVA F(3,12)=14.62, P-value=0.0036, N=3 independent MAC differentiations/transient transfection with siRNA per group, followed by Dunnett's post-hoc tests: $ES_{40KD-SCR}$=-0.1502 [-0.4509, 0.1504], P-$value_{40KO-WT}$=0.3576; $ES_{41KD-SCR}$=-0.5257 [-0.8264, -0.2250], P-$value_{41KD-SCR}$=0.0041; $ES_{DKD-SCR}$=-0.5075 [-0.8082, -0.2069], P-$value_{DKD-SCR}$=0.0048.

Effect sizes (ES) are reported as unstandardized point estimates with 95% confidence intervals in the same unit as depicted on each graph.

Supplementary Figure 4. Expression of lipid and lysosomal clearance genes in human iPSC-derived microglia (iMGLs) lacking *BHLHE40* and/or *BHLHE41*. Expression of lipid and lysosomal clearance genes measured by RT-qPCR, N=5/group. log2(fold change) (log2FC) is calculated with WT iMGLs as reference. Differences of

means between groups were tested using one-way ANOVA with repeated measures followed by Dunnett's post-hoc test.

ABCA1: ANOVA F(3,12)=3.17, P-value=0.0640 followed by Dunnett's post-hoc tests: $ES_{40KO\text{-}WT}$=1.51 [-0.1786, 3.207], $P\text{-value}_{40KO\text{-}WT}$=0.0822; $ES_{41KO\text{-}WT}$=1.102 [-0.5912, 2.794], $P\text{-value}_{41KO\text{-}WT}$=0.2408; $ES_{DKO\text{-}WT}$=1.813 [0.1202, 3.506], $P\text{-value}_{DKO\text{-}WT}$=0.0356.

ABCG1: ANOVA F(3,12)=1.45, P-value=0.2783 followed by Dunnett's post-hoc tests: $ES_{40KO\text{-}WT}$=0.8994 [-0.6879, 2.487], $P\text{-value}_{40KO\text{-}WT}$=0.3357; $ES_{41KO\text{-}WT}$=0.6468 [-0.9405, 2.234], $P\text{-value}_{41KO\text{-}WT}$=0.5775; $ES_{DKO\text{-}WT}$=1.178 [-0.4097, 2.765], $P\text{-value}_{DKO\text{-}WT}$=0.1637.

APOE: ANOVA F(3,12)=3.89, P-value=0.0372 followed by Dunnett's post-hoc tests: $ES_{40KO\text{-}WT}$=1.546 [0.3100, 2.782], $P\text{-value}_{40KO\text{-}WT}$=0.0149; $ES_{41KO\text{-}WT}$=0.9622 [-0.2740, 2.198], $P\text{-value}_{41KO\text{-}WT}$=0.1395; $ES_{DKO\text{-}WT}$=0.6654 [-0.5708, 1.902], $P\text{-value}_{DKO\text{-}WT}$=0.3732.

CLU: ANOVA F(3,12)=0.7083, P-value=0.5654 followed by Dunnett's post-hoc tests: $ES_{40KO\text{-}WT}$=0.7216 [-4.371, 5.814], $P\text{-value}_{40KO\text{-}WT}$=0.9633; $ES_{41KO\text{-}WT}$=2.306 [-2.786, 7.399], $P\text{-value}_{41KO\text{-}WT}$=0.5016; $ES_{DKO\text{-}WT}$=2.189 [-2.903, 7.282], $P\text{-value}_{DKO\text{-}WT}$=0.5395.

CTSB: ANOVA F(3,12)=2.620, P-value=0.0988 followed by Dunnett's post-hoc tests: $ES_{40KO\text{-}WT}$=0.8508 [-0.099, 1.801], $P\text{-value}_{40KO\text{-}WT}$=0.0818; $ES_{41KO\text{-}WT}$=0.6270 [-0.3231, 1.577], $P\text{-value}_{41KO\text{-}WT}$=0.2319; $ES_{DKO\text{-}WT}$=0.8646 [-0.0548, 1.815], $P\text{-value}_{DKO\text{-}WT}$=0.0764.

CTSZ: ANOVA F(3,12)=4.201, P=0.0301 followed by Dunnett's post-hoc tests: $ES_{40KO\text{-}WT}$=0.7712 [0.0751, 1.467], $P\text{-value}_{40KO\text{-}WT}$=0.0298; $ES_{41KO\text{-}WT}$=0.6248 [-0.0713, 1.321], $P\text{-value}_{41KO\text{-}WT}$=0.0810; $ES_{DKO\text{-}WT}$=0.8092 [0.1131, 1.505], $P\text{-value}_{DKO\text{-}WT}$=0.0229.

FABP5: ANOVA F(3,12)=3.720, P-value=0.0422 followed by Dunnett's post-hoc tests: $ES_{40KO\text{-}WT}$=0.5012 [0.0895, 0.9129], $P\text{-value}_{40KO\text{-}WT}$=0.0175; $ES_{41KO\text{-}WT}$=0.2558 [-0.1559, 0.6675], $P\text{-value}_{41KO\text{-}WT}$=0.2712; $ES_{DKO\text{-}WT}$=0.3398 [-0.0719, 0.7515], $P\text{-value}_{DKO\text{-}WT}$=0.1129.

LPL: ANOVA F(3,12)=7.025, P-value=0.0056 followed by Dunnett's post-hoc tests: $ES_{40KO\text{-}WT}$=1.207 [0.3591, 2.054], $P\text{-value}_{40KO\text{-}WT}$=0.0065; $ES_{41KO\text{-}WT}$=0.9020 [0.0544, 1.750], $P\text{-value}_{41KO\text{-}WT}$=0.0368; $ES_{DKO\text{-}WT}$=1.296 [0.4487, 2.144], $P\text{-value}_{DKO\text{-}WT}$=0.0039.

NR1H3: ANOVA F(3,12)=3.438, P-value=0.0520 followed by Dunnett's post-hoc tests: $ES_{40KO-WT}$=1.129 [0.001, 2.258], $P\text{-value}_{40KO-WT}$=0.0500; $ES_{41KO-WT}$=0.9856 [-0.1442, 2.115], $P\text{-value}_{41KO-WT}$=0.0910; ES_{DKO-WT}=1.166 [0.0364, 2.296], $P\text{-value}_{DKO-WT}$=0.0429.
SPP1: ANOVA F(3,12)=0.0833, P-value=0.9678 followed by Dunnett's post-hoc tests: $ES_{40KO-WT}$=-0.0308 [-1.027, 0.9655], $P_{40KO-WT}$=0.9996; $ES_{41KO-WT}$=0.1156 [-0.8807, 1.112], $P\text{-value}_{41KO-WT}$=0.9790; ES_{DKO-WT}=0.1126 [-0.8837, 1.109], $P\text{-value}_{DKO-WT}$=0.9805.
TREM2: ANOVA F(3,12)=4.113, P-value=0.0320 followed by Dunnett's post-hoc tests: $ES_{40KO-WT}$=0.7654 [0.1746, 1.356], $P\text{-value}_{40KO-WT}$=0.0120; $ES_{41KO-WT}$=0.4536 [-0.1372, 1.044], $P\text{-value}_{41KO-WT}$=0.1462; ES_{DKO-WT}=0.4688 [-0.1220, 1.060], $P\text{-value}_{DKO-WT}$=0.1303.

Effect sizes (ES) are reported as unstandardized point estimates with 95% confidence intervals in the same unit as depicted on each graph.

Supplementary Figure 5. LXR-dependent accumulation of lipid droplets (LDs) in human iPSC-derived microglia (iMGLs) lacking BHLHE40 and/or BHLHE41.

A. LD content (BODIPY gMFI) measured by flow cytometry in BODIPY-positive WT iMGLs upon treatment with an LXR agonist (TO901317, 10uM, 48h) compared to vehicle control (Veh, DMSO). Differences of means between groups were tested using the paired t.test. t=7.049, df=2, P-value=0.0195, N=3 independent iMGLs differentiations per group $ES_{LXR\_agonist-Veh}$=711.3 [277.2, 1145]
B. LD content (BODIPY gMFI) measured by flow cytometry in BODIPY-positive WT, 40KO, 41KO, and DKO iMGLs treated with an LXR antagonist (GSK2033, 2uM, 24h) compared to vehicle control (Veh, DMSO). Differences of means between groups were tested using two-way ANOVA Genotype: F(3,8)=34.17, P<0.0001, Treatment: F(1,8)=152.9, P-value<0.0001, Genotype x Treatment F(3,8) = 40.04, P-value<0.0001, N=2 independent iMGLs differentiation followed by Dunnett's post-hoc tests Veh: $ES_{40KO-WT}$=2999 [2325, 3672], $P\text{-value}_{40KO-WT}$<0.0001; $ES_{41KO-WT}$=1959 [1286, 2632], $P\text{-value}_{41KO-WT}$<0.0001; ES_{DKO-WT}=2508 [1835, 3181], $P\text{-value}_{DKO-WT}$<0.0001; LXR antagonist: $ES_{40KO-WT}$=-268.2 [-941.1, 405.1], P-

value$_{40KO-WT}$=0.5531; ES$_{41KO-WT}$=-265.4 [-938.6, 407.8], P-value$_{41KO-WT}$=0.5602; ES$_{DKO-WT}$=887.1 [213.8, 1560], P-value$_{DKO-WT}$=0.0133

Data plotted as mean ± SEM. 40KO = BHLHE40 KO iMGLs, 41KO = BHLHE41 KO iMGLs, DKO = BHLHE40 and BHLHE41 double KO iMGLs, WT = iMGLs derived from the parental iPSC line. Effect sizes (ES) are reported as unstandardized point estimates with 95% confidence intervals in the same unit as depicted on each graph

Supplementary Figure 6. Reduced secretion of proinflammatory cytokines associated with complete loss (KO) or reduced levels (KD) of BHLHE40 and/or BHLHE41 in human iPSC-derived microglia (iMGLs) and THP-1 macrophages (MACs). Quantifications and dot blots of cytokines secreted in conditioned media from (A and B) human iPSC-derived microglia (iMGLs) with genetic inactivation of BHLHE40 and/or BHLHE41 or (C and D) human THP-1 macrophages (MACs) treated with BHLHE40 and/or BHLHE41 siRNAs measured using the Proteome Profiler Human Cytokine Array Kit (R&D Systems).

A. Levels of cytokines secreted in conditioned media quantified as target density (i.e. mean target spot intensity multiplied by target spot area) normalized to reference density (i.e. mean reference spot intensity multiplied by reference spot area). Data are plotted as log2(fold change) which is calculated with WT iMGLs. Differences of means between groups were tested using one-way ANOVA with repeated measures followed by Dunnett's post-hoc test. N=3/group

CXCL12: ANOVA F(3,6)=2.42, P-value=0.1645, followed by Dunnett's post-hoc tests: ES$_{40KO-WT}$=-0.0181 [-0.2717, 0.2353], P-value$_{40KO-WT}$=0.9916; ES$_{41KO-WT}$=-0.0817 [-0.3352, 0.1718], P-value$_{41KO-WT}$=0.6498; ES$_{DKO-WT}$=-0.1990 [-0.4525, 0.0545], P-value$_{DKO-WT}$=0.1163.

ICAM1: ANOVA F(3,6)=0.745, P-value=0.5634, followed by Dunnett's post-hoc tests: ES$_{40KO-WT}$=0.0361 [-0.2259, 0.2981], P-value$_{40KO-WT}$=0.9482; ES$_{41KO-WT}$=-0.0817 [-0.3438, 0.1802], P-value$_{41KO-WT}$=0.6690; ES$_{DKO-WT}$=-0.1496 [-0.2470, 0.2769], P-value$_{DKO-WT}$=0.9957.

IL13: ANOVA $F(3,6)=4.534$, P-value=0.0550, followed by Dunnett's post-hoc tests: $ES_{40KO-WT}=-0.2847$ [-0.5838, -0.00143], $P\text{-value}_{40KO-WT}=0.0501$; $ES_{41KO-WT}=-0.2756$ [-0.5747, -0.0023], $P\text{-value}_{41KO-WT}=0.0500$; $ES_{DKO-WT}=-0.3076$ [-0.6067, -0.0085], $P\text{-value}_{DKO-WT}=0.0448$.

IL16: ANOVA $F(3,6)=2.354$, P-value=0.1712, followed by Dunnett's post-hoc tests: $ES_{40KO-WT}=-0.2548$ [-0.5799, 0.0701], $P\text{-value}_{40KO-WT}=0.1168$; $ES_{41KO-WT}=-0.2251$ [-0.5501, 0.0999], $P\text{-value}_{41KO-WT}=0.1685$; $ES_{DKO-WT}=-0.1566$ [-0.4817, 0.1684], $P\text{-value}_{DKO-WT}=0.3817$.

IL8: ANOVA $F(3,3)=2.410$, P-value=0.2445, followed by Dunnett's post-hoc tests: $ES_{40KO-WT}=-0.2953$ [-0.7676, 0.1770], $P\text{-value}_{40KO-WT}=0.1553$; $ES_{41KO-WT}=-0.1517$ [-0.6240, 0.3206], $P\text{-value}_{41KO-WT}=0.4876$; $ES_{DKO-WT}=-0.1172$ [-0.5895, 0.3550], $P\text{-value}_{DKO-WT}=0.6382$.

MIF: ANOVA $F(3,6)=2.065$, P-value=0.2064, followed by Dunnett's post-hoc tests: $ES_{40KO-WT}=-0.2606$ [-0.7211, 0.1999], $P\text{-value}_{40KO-WT}=0.2776$; $ES_{41KO-WT}=-0.3457$ [-0.8062, -0.0011], $P\text{-value}_{41KO-WT}=0.0491$; $ES_{DKO-WT}=-0.1342$ [-0.5947, 0.3263], $P\text{-value}_{DKO-WT}=0.7083$.

SERPINE1: ANOVA $F(3,6)=1.079$, P-value=0.4264, followed by Dunnett's post-hoc tests: $ES_{40KO-WT}=-0.2159$ [-0.6675, 0.2357], $P\text{-value}_{40KO-WT}=0.3872$; $ES_{41KO-WT}=-0.2367$ [-0.6883, 0.2150], $P\text{-value}_{41KO-WT}=0.3263$; $ES_{DKO-WT}=-0.1515$ [-0.6031, 0.3001], $P\text{-value}_{DKO-WT}=0.6255$.

CCL2: ANOVA $F(3,6)=6.951$, P-value=0.0223, followed by Dunnett's post-hoc tests: $ES_{40KO-WT}=-0.2371$ [-0.4208, -0.0533], $P\text{-value}_{40KO-WT}=0.0173$; $ES_{41KO-WT}=-0.2109$ [-0.3946, -0.0271], $P\text{-value}_{41KO-WT}=0.0288$; $ES_{DKO-WT}=-0.0887$ [-0.2714, 0.0961], $P\text{-value}_{DKO-WT}=0.3887$.

IL18: ANOVA $F(3,6)=2.551$, P-value=0.1129, followed by Dunnett's post-hoc tests: $ES_{40KO-WT}=-0.2271$ [-0.4208, 0.0633], $P\text{-value}_{40KO-WT}=0.1067$; $ES_{41KO-WT}=-0.1909$ [-0.3946, 0.0471], $P\text{-value}_{41KO-WT}=0.1175$; $ES_{DKO-WT}=-0.1887$ [-0.2714, 0.00961], $P\text{-value}_{DKO-WT}=0.0763$.

IL1RA: ANOVA $F(3,6)=2.032$, P-value=0.2109, followed by Dunnett's post-hoc tests: $ES_{40KO-WT}=-0.1429$ [-0.4931, 0.2073], $P\text{-value}_{40KO-WT}=0.4957$; $ES_{41KO-WT}=-0.1878$ [-

0.5380, 0.1624], P-value$_{41KO-WT}$=0.3114; ES$_{DKO-WT}$=-0.2722 [-0.6224, -0.0077], P-value$_{DKO-WT}$=0.0493.

C. Levels of cytokines secreted in conditioned media quantified as target density (i.e. mean target spot intensity multiplied by target spot area) normalized to reference density (i.e. mean reference spot intensity multiplied by reference spot area). log2(fold change) is calculated with SCR MACs as reference. Differences of means between groups were tested using one-way ANOVA with repeated measures followed by Dunnett's post-hoc test. N=3/group

MIP1α: ANOVA F(3,6)=5.935, P-value=0.0315, followed by Dunnett's post-hoc tests: ES$_{40KD-SCR}$=-0.2016 [-0.3663, -0.0368], P-value$_{40KD-SCR}$=0.0219; ES$_{41KD-SCR}$=-0.1603 [-0.3250, 0.0044], P-value$_{41KD-SCR}$=0.0555; ES$_{DKD-SCR}$=-0.0632 [-0.2279, 0.1015], P-value$_{DKD-SCR}$=0.5377.

MIF: ANOVA F(3,6)=6.201, P-value=0.0287, followed by Dunnett's post-hoc tests: ES$_{40KD-SCR}$=-0.2372 [-0.4769, 0.00257], P-value$_{40KD-SCR}$=0.0521; ES$_{41KD-SCR}$=-0.2244 [-0.4641, 0.0152], P-value$_{41KD-SCR}$=0.0640; ES$_{DKD-SCR}$=-0.3173 [-0.5570, -0.0773], P-value$_{DKD-SCR}$=0.0154.

IL8: ANOVA F(3,6)=2.734, P-value=0.1361, followed by Dunnett's post-hoc tests: ES$_{40KD-SCR}$=-0.1798 [-0.3958, 0.0363], P-value$_{40KD-SCR}$=0.0965; ES$_{41KD-SCR}$=-0.0678 [-0.2840, 0.1482], P-value$_{41KD-SCR}$=0.6653; ES$_{DKD-SCR}$=-0.1504 [-0.3665, 0.0655], P-value$_{DKD-SCR}$=0.1661.

IL1RA: ANOVA F(3,6)=32.74, P-value=0.0004, followed by Dunnett's post-hoc tests: ES$_{40KD-SCR}$=-0.5182 [-0.7117, -0.3246], P-value$_{40KD-SCR}$=0.0004; ES$_{41KD-SCR}$=-0.5519 [-0.7454, -0.3583], P-value$_{41KD-SCR}$=0.0003; ES$_{DKD-SCR}$=-0.3719 [-0.5655, -0.1783], P-value$_{DKD-SCR}$=0.0025.

IL1β: ANOVA F(3,6)=2.989, P-value=0.1177, followed by Dunnett's post-hoc tests: ES$_{40KD-SCR}$=-0.2059 [-0.9746, 0.5628], P-value$_{40KD-SCR}$=0.7523; ES$_{41KD-SCR}$=-0.1785 [-0.9472, 0.5902], P-value$_{41KD-SCR}$=0.8159; ES$_{DKD-SCR}$=-0.7064 [-1.475, 0.0623], P-value$_{DKD-SCR}$=0.0684.

ICAM1: ANOVA F(3,6)=14.42 P-value=0.0038, followed by Dunnett's post-hoc tests: ES$_{40KD-SCR}$=-0.1981 [-0.2943, -0.1019], P-value$_{40KD-SCR}$=0.0017; ES$_{41KD-SCR}$=-0.1339 [-

0.2301, -0.0375], P-$value_{41KD\text{-}SCR}$=0.0122; $ES_{DKD\text{-}SCR}$=-0.1339 [-0.2301, -0.0377], P-$value_{DKD\text{-}SCR}$=0.0122.

CXCL11: ANOVA F(3,6)=4.333 P-value=0.0601, followed by Dunnett's post-hoc tests: $ES_{40KD\text{-}SCR}$=-0.1663 [-0.3380, 0.0054], P-$value_{40KD\text{-}SCR}$=0.0565; $ES_{41KD\text{-}SCR}$=-0.1148 [-0.2865, 0.0568], P-$value_{41KD\text{-}SCR}$=0.1856; $ES_{DKD\text{-}SCR}$=-0.1790 [-0.3508, -0.0072], P-$value_{DKD\text{-}SCR}$=0.0425.

CCL5: ANOVA F(3,6)=15.64 P-value=0.0031, followed by Dunnett's post-hoc tests: $ES_{40KD\text{-}SCR}$=-0.1876 [-0.2799, -0.0953], P-$value_{40KD\text{-}SCR}$=0.0018; $ES_{41KD\text{-}SCR}$=-0.1615 [-0.2537, -0.0692], P-$value_{41KD\text{-}SCR}$=0.0040; $ES_{DKD\text{-}SCR}$=-0.1293 [-0.2216, -0.0371], P-$value_{DKD\text{-}SCR}$=0.0119.

TNFα: ANOVA F(3,6)=93.00 P-value<0.0001, followed by Dunnett's post-hoc tests: $ES_{40KD\text{-}SCR}$=-0.6826 [-0.9495, -0.4157], P-$value_{40KD\text{-}SCR}$=0.0005; $ES_{41KD\text{-}SCR}$=-0.7145 [-0.9814, -0.4476], P-$value_{41KD\text{-}SCR}$=0.0004; $ES_{DKD\text{-}SCR}$=-1.438 [-1.705, -1.171], P-$value_{DKD\text{-}SCR}$<0.0001.

SERPINE1: ANOVA F(3,6)=8.744 P-value=0.0131, followed by Dunnett's post-hoc tests: $ES_{40KD\text{-}SCR}$=-0.2327 [-0.3997, -0.0657], P-$value_{40KD\text{-}SCR}$=0.0122; $ES_{41KD\text{-}SCR}$=-0.0641 [-0.2312, 0.1028], P-$value_{41KD\text{-}SCR}$=0.5366; $ES_{DKD\text{-}SCR}$=-0.2097 [-0.3766, -0.0427], P-$value_{DKD\text{-}SCR}$=0.0196.

Effect sizes (ES) are reported as unstandardized point estimates with 95% confidence intervals in the same unit as depicted on each graph
