# Supplementary material for: BHLHE40/41 regulate microglia and peripheral macrophage responses associated with Alzheimer’s disease and other disorders of lipid-rich tissues
Source: Nat Commun. 2024 Mar 6;15:2058. doi: 10.1038/s41467-024-46315-7 (PMC10917780; doi:10.1038/s41467-024-46315-7)
Supplement: Supplementary file 3 — Description of Additional Supplementary Information [file 41467_2024_46315_MOESM3_ESM.pdf]

Description of Additional Supplementary Files

File Name: Supplementary Dataset 1

Description: DLAM_datasets sheet. Human and mouse disease-associated microglia/lipid-associated macrophage (DLAM) sc/snRNA-seq datasets used to reconstruct gene regulatory networks.

Individual_DLAM_TF_mouse sheet. Mouse DLAM TFs nominated by individual network (dataset) before meta-analysis (listed in alphabetical order)

Individual_DLAM_TF_human sheet. Human DLAM TFs nominated by individual network (dataset) before meta-analysis (listed in alphabetical order)

DLAM_TFs sheet. Candidate human and mouse DLAM TFs nominated in at least half human and mouse GRNs, respectively (listed in alphabetical order).

DLAM_genesets sheet. Human and mouse DLAM genesets used in regulon enrichment, RRHO, GSEA, and other analyses (genes listed in alphabetical order).

File Name: Supplementary Dataset 2

Description: BHLHE40/41_regulons sheet. BHLHE40 and BHLHE41 human and mouse regulons from meta-analysed human and mouse GRNs. Meta-analyzed GRNs were generated by aggregating all the bootstraps from individual networks generated by ARACNe.

LAM_and_DAM_genes_in_BHLHE40/41_regulons sheet. Human and mouse LAM and DAM genes in BHLHE40 and BHLHE41 human and mouse regulons (see BHLHE40/41_regulons sheet). Human LAM genes in BHLHE40 and BHLHE41 human regulons. Human LAM genes were selected from Jaitin *et al*. [6] (Dataset S6, FDR Adj.P-value < 0.05). Mouse DAM genes in Bhlhe40 and Bhlhe41 mouse regulons. Mouse DAM genes were selected from Keren-Shaul *et al*. [8] (Table S2).

LAM_and_DAM_genes_with_promoter_proxy-bound_by_BHLHE40/41 sheet. Human and mouse LAM and DAM genes with promoters proxy-bound by BHLHE40 and/or BHLHE41. LAM genes were selected from Jaitin *et al*. [6] (Dataset S6, FDR Adj.P-value < 0.05). Mouse DAM genes in Bhlhe40 and Bhlhe41 mouse regulons. Mouse DAM genes were selected from Keren-Shaul *et al*. [8] (Table S2).

Genes_in_most_significant-RRHO_overlaps sheet. Genes in most significant overlaps from rank-rank hypergeometric overlap (RRHO) analyses. Z scores (Z.std) from RNA-seq differential

gene expression analyses were assigned to each overlapping gene to run Ingenuity Pathway Analysis (IPA). Human LAM signature genes in Figure 4A and Supplementary Figure 7A were selected from Jaitin *et al.* [6] (Dataset S6, FDR Adj.P-value < 0.05). Mouse DAM signature genes in Figure 7A were selected from Keren-Shaul *et al.* [8] (Table S3, FDR Adj.P-value < 0.05).

File Name: Supplementary Dataset 3

Description: Differential gene expression (DGEA) and gene set enrichment (GSEA) analysis of *BHLHE40* and/or *BHLHE41* knockout (KO) in human iPSC-derived microglia (iMGLs). 40KO = BHLHE40 KO iMGLs, 41KO = BHLHE41 KO iMGLs, DKO = BHLHE40 and BHLHE41 double KO iMGLs, WT = iMGLs derived from the parental iPSC line.

File Name: Supplementary Dataset 4.

Description: Differential gene expression (DGEA) and gene set enrichment (GSEA) analysis of *BHLHE40* and/or *BHLHE41* knockdown (KD) in human THP-1 macrophages (MACs). 40KD = MACs treated with BHLHE40 siRNA, 41KD = MACs treated with BHLHE41 siRNA, DKD = MACs treated with BHLHE40 and BHLHE41 siRNA, SCR = MACs treated with scrambled siRNA.

File Name: Supplementary Dataset 5

Description: Differential gene expression (DGEA) and gene set enrichment (GSEA) analysis of *Bhlhe40* and *Bhlhe41* double knockout (DKO) in mouse microglia. DKO = Bhlhe40/41 DKO mouse microglia, compared to microglia derived from wild-type control mice.

File Name: Supplementary Dataset 6

Description: Sequences of primers used for RT-qPCR and sequences of guide RNAs, single-stranded oligodeoxynucleotides (ssODNs), and PCR primers used for CRISPR/Cas9-mediated HDR.
