# Supplementary material for: BHLHE40/41 regulate microglia and peripheral macrophage responses associated with Alzheimer’s disease and other disorders of lipid-rich tissues
Source: Nat Commun. 2024 Mar 6;15:2058. doi: 10.1038/s41467-024-46315-7 (PMC10917780; doi:10.1038/s41467-024-46315-7)
Supplement: Supplementary file 10 — Reporting Summary [file 41467_2024_46315_MOESM10_ESM.pdf]

nature portfolio

Corresponding author(s): Alison Goate, D.Phil
Edoardo Marcora, PhD

Last updated by author(s): Jan 29, 2024

Reporting Summary

Nature Portfolio wishes to improve the reproducibility of the work that we publish. This form provides structure for consistency and transparency in reporting. For further information on Nature Portfolio policies, see our Editorial Policies and the Editorial Policy Checklist.

Statistics

For all statistical analyses, confirm that the following items are present in the figure legend, table legend, main text, or Methods section.

n/a	Confirmed	
☐	☒	The exact sample size (*n*) for each experimental group/condition, given as a discrete number and unit of measurement
☐	☒	A statement on whether measurements were taken from distinct samples or whether the same sample was measured repeatedly
☐	☒	The statistical test(s) used AND whether they are one- or two-sided<br>*Only common tests should be described solely by name; describe more complex techniques in the Methods section.*
☒	☐	A description of all covariates tested
☐	☒	A description of any assumptions or corrections, such as tests of normality and adjustment for multiple comparisons
☐	☒	A full description of the statistical parameters including central tendency (e.g. means) or other basic estimates (e.g. regression coefficient) AND variation (e.g. standard deviation) or associated estimates of uncertainty (e.g. confidence intervals)
☐	☒	For null hypothesis testing, the test statistic (e.g. *F*, *t*, *r*) with confidence intervals, effect sizes, degrees of freedom and *P* value noted<br>*Give P values as exact values whenever suitable.*
☒	☐	For Bayesian analysis, information on the choice of priors and Markov chain Monte Carlo settings
☒	☐	For hierarchical and complex designs, identification of the appropriate level for tests and full reporting of outcomes
☐	☒	Estimates of effect sizes (e.g. Cohen's *d*, Pearson's *r*), indicating how they were calculated

Our web collection on statistics for biologists contains articles on many of the points above.

Software and code

Policy information about availability of computer code

Data collection	Flow cytometry: Attune Flow Cytometer (Thermo Fisher Scientific)<br>RT-qPCR: QuantStudio 7.0 (Applied Biosystems)<br>Western blotting, Proteome Profiler: iBright (Applied Biosystems) or UVP ChemiDoc-ItTS2 Imager (UVP)<br>Cholesterol efflux, ELISA: Varioskan LUX multimode microplate reader (Thermo Fisher Scientific)
Data analysis	Data representation and analysis: Prism 9.3.1 (GraphPad Software, Inc)<br>Rank-rank hypergeometric overlap (RRHO): RRHO2 package was used https://rdrr.io/github/RRHO2/RRHO2/f/vignettes/RRHO2.Rmd<br>Pathway analysis: Ingenuity Pathway Analysis Qiagen Inc., (https://digitalinsights.qiagen.com/IPA)<br>Flow cytometry: FCS Express 7 Research (version 7.12.0007 (Mac)) (De Novo Software by Dotmatics) and BD FACSDiva 4.0<br>Bulk RNA sequencing:<br>a) Raw reads were mapped to the mouse genome (mm10) or human genome hg38 using STAR v2.5.3a.<br>b) Differential gene expression analysis was performed using a linear mixed model implemented in dream (differential expression for repeated measures, variancePartition R package v1.23.1 and R v3.5.3<br>Custom code developed to perform gene regulatory network analysis: https://github.com/marcoralab/bhlhe_manuscript

For manuscripts utilizing custom algorithms or software that are central to the research but not yet described in published literature, software must be made available to editors and reviewers. We strongly encourage code deposition in a community repository (e.g. GitHub). See the Nature Portfolio guidelines for submitting code & software for further information.

Data

Policy information about availability of data

All manuscripts must include a data availability statement. This statement should provide the following information, where applicable:

- Accession codes, unique identifiers, or web links for publicly available datasets
- A description of any restrictions on data availability
- For clinical datasets or third party data, please ensure that the statement adheres to our policy

The accession numbers for sc/sn-RNASeq data used to reconstruct gene regulatory network are listed in Supplementary Table 1:
GSE127892
GSE127884
GSE129788
GSE130626
GSE128518
GSE97310
GSE123587
GSE123587
GSE136103
GSE129516
Synapse (syn18485175)
Synapse (syn21125841)
Synapse (syn21438358)
GSE137444
GSE128518
GSE115469
GSE136103

Raw bulk RNA sequencing data generated in this study are available in GEO
a) iPSC-derived microglia lacking BHLHE40 (BHLHE-KO), BHLHE41 (BHLHE41-KO) or both of them BHLHE40/41 (BHLHE40/41-DKO) along with isogenic wild type (WT) control GSE253943
b) THP-1 macrophages with partial reduction of BHLHE40 (BHLHE40-KD), BHLHE41 (BHLHE41-KD), or both of them BHLHE40/41 (BHLHE40/41-DKD) along with scrambled control (SCR) GSE253992
c) Acutely isolated microglia from mice lacking Bhlhe40/41 (Bhlhe40/41-DKO) along with WT control GSE254233

Research involving human participants, their data, or biological material

Policy information about studies with human participants or human data. See also policy information about sex, gender (identity/presentation), and sexual orientation and race, ethnicity and racism.

Reporting on sex and gender	This study does not include human participants
Reporting on race, ethnicity, or other socially relevant groupings	This study does not include human participants
Population characteristics	This study does not include human participants
Recruitment	This study does not include human participants
Ethics oversight	This study does not include human participants

Note that full information on the approval of the study protocol must also be provided in the manuscript.

Field-specific reporting

Please select the one below that is the best fit for your research. If you are not sure, read the appropriate sections before making your selection.

☒ Life sciences ☐ Behavioural & social sciences ☐ Ecological, evolutionary & environmental sciences

For a reference copy of the document with all sections, see nature.com/documents/nr-reporting-summary-flat.pdf

Life sciences study design

All studies must disclose on these points even when the disclosure is negative.

Sample size	Sample size for iPSC-derived microglia, THP-1 macrophages and mouse microglia were estimated using pwr (version 1.3-0) in R. Briefly, two

Sample size	preliminary experiments per assay were performed. Based on preliminary experiments for each assay, Cohen's d effect size was calculated. N (number of observations per group) was calculated using pwr.anova.test (k = 4 (number of groups), f = Cohen's d for each assay, sig.level = 0.05, power = 0.8). N was estimated for 5. We aimed to have N=5-6 independent iPSC-derived microglia differentiation and THP-1 macrophage transient transfections per groups in every assay we performed. We assumed same sample size for bulk RNAseq analysis using preliminary power analysis from RT-qPCR as a justification.
Data exclusions	No data were excluded for analysis
Replication	All presented data are representative of the same experiment performed in at least 3 independent repetition (i.e. independent iPSC-derived microglia differentiation or independent transient transfection of THP-1 macrophages). All attempts at replication were successful. Th exact number of rpelication is clearly stated in each Figure legend. Findings from iPSC-derived microglia (transcriptional and functional changes) were replicated in another cellular model (THP-1 macrophages with reduced expression of BHLHE40 and /or BHLHE41).
Randomization	For in vivo experiments, animals were randomly assigned to experimental groups. For in vitro studies separate iMGL wells and THP-1 macrophage wells were randomly assigned to functional experiments.
Blinding	Experimentators were not blind to the genotype in this study. All the functional experiments were mainly performed by one experimentalist.

Reporting for specific materials, systems and methods

We require information from authors about some types of materials, experimental systems and methods used in many studies. Here, indicate whether each material, system or method listed is relevant to your study. If you are not sure if a list item applies to your research, read the appropriate section before selecting a response.

Materials & experimental systems

n/a	Involved in the study
☐	☒ Antibodies
☐	☒ Eukaryotic cell lines
☒	☐ Palaeontology and archaeology
☐	☒ Animals and other organisms
☒	☐ Clinical data
☒	☐ Dual use research of concern
☒	☐ Plants

Methods

n/a	Involved in the study
☒	☐ ChIP-seq
☐	☒ Flow cytometry
☒	☐ MRI-based neuroimaging

Antibodies

Antibodies used

Rabbit anti-BHLHE40 (Thermo Fisher Scientific, PA5-83044)
Rabbit anti-BHLHE41 antibody (Biorbyt, orb224120)
Mouse anti-ABCA1 antibody (Abcam, 18180)
Goat anti-APOE antibody (Millipore, AB947)
Mouse anti-Actin antibody (Sigma-Aldrich, A5441)
HRP Goat anti-rabbit IgG antibody (Peroxidase) (Vector Laboratories PI-1000)
HRP Horse anti-mouse IgG antibody (Peroxidase) (Vector Laboratories PI-2000)
Rabbit anti-goat IgG secondary antibody, HRP conjugated (Invitrogen, 61-1620)
Rat anti-CD38-PE (Miltenyi, 130-103-008),
Recombinant human IgG1 anti-Ly6G-APC (Miltenyi, 130-120-803),
Rat anti-Ly6C-BV510 (Biolegend, 127633),
Rat anti-CD11b-FITC (Biolegend, 101206),
Rat anti-MHCII-BV421 (Biolegend, 107632),
Mouse anti-CD45.2-PE-Cy7 (Biolegend, 109830),

Validation

BHLHE40 and BHLHE41 antibodies were KO validated in our studies (western blot).
ABCA1 antibody was validated using LXR agonist T0901317 (10uM, 24h) (western blot)
For APOE, secondary antibodies (HRP conjugated), and flow cytometry antibodies, validation studies can be found on the manufacturer's website.

APOE: https://www.emdmillipore.com/US/en/product/Anti-Apolipoprotein-E-Antibody,MM_NF-AB947?ReferrerURL=https%3A%2F%2Fwww.google.com%2F
Actin: https://www.thermofisher.com/antibody/product/beta-Actin-Antibody-clone-15G5A11-E2-Monoclonal/MA1-140
HRP goat anti-rabbit: https://vectorlabs.com/products/peroxidase-goat-anti-rabbit-igg
HRP horse anti-mouse: https://vectorlabs.com/products/peroxidase-horse-anti-mouse-igg
rabbit anti-goat: https://www.thermofisher.com/antibody/product/Rabbit-anti-Goat-IgG-H-L-Secondary-Antibody-Polyclonal/61-1620
Ly6G-APC: https://www.miltenyibiotec.com/US-en/products/ly-6g-antibody-anti-mouse-reafinity-rea526.html#conjugate=apc:size=30-ug-in-200-ul
CD38-PE: https://www.miltenyibiotec.com/US-en/products/cd38-antibody-anti-mouse-90-4.html#conjugate=pe:size=9-ug-in-300-ul
Ly6C-BV510: https://www.biolegend.com/ja-jp/products/brilliant-violet-510-anti-mouse-ly-6g-antibody-9121?GroupID=BLG7232

CD11b-FITC: https://www.biolegend.com/en-ie/products/fitc-anti-mouse-human-cd11b-antibody-347?GroupID=BLG10660
MHCII-BV421: https://www.biolegend.com/en-ie/products/brilliant-violet-421-anti-mouse-i-a-i-e-antibody-7147
CD45.2-PE-Cy7: https://www.biolegend.com/en-ie/products/pe-cyanine7-anti-mouse-cd45-2-antibody-4918

Eukaryotic cell lines

Policy information about cell lines and Sex and Gender in Research

Cell line source(s)	THP-1 human acute monocytic leukemia cell line (RRID:CVCL_0006) (derived from male)<br>WTC-11 donor iPSC line (derived from male) Coriell Institute for Medical Research, GM25256
Authentication	Cell lines were not authenticated but the BHLHE40 and BHLHE41 levels of expression (RT-qPCR and WB) were frequently validated.
Mycoplasma contamination	All cell lines were negative for mycoplasma contamination
Commonly misidentified lines<br>(See ICLAC register)	This study does not use commonly misidentified cell lines

Animals and other research organisms

Policy information about studies involving animals; ARRIVE guidelines recommended for reporting animal research, and Sex and Gender in Research

Laboratory animals	All mice used in this study were maintained on the C57BL/6J genetic background, WT C57BL/6J mice were obtained from Janvier Labs or bred in-house. Mice were bred and maintained at Comparative Medicine Biomedicum facility of Karolinska Institutet (Stockholm, Sweden). All animal experiments were carried out according to valid project licenses, which were approved and regularly controlled by Swedish Veterinary Authorities. Mice analyzed in this study were 6 month old (first batch, N=3/ group) and 2 month old (second batch, N=3/group). Dark/Light cycle (12h/12h), ambient temperature and humidity were centrally regulated and animals were closely monitored by resident veterinarians for wellbeing.
Wild animals	This study did not involved wild type animals
Reporting on sex	Only females were used in this study.
Field-collected samples	This study did not involved field-collected samples
Ethics oversight	Mice were bred and maintained at the Comparative Medicine Biomedicum facility of Karolinska Institutet (Stockholm, Sweden). All mouse experiments were performed according to valid project licenses, which are approved and regularly controlled by the Swedish Veterinary Authorities.

Note that full information on the approval of the study protocol must also be provided in the manuscript.

Plants

Seed stocks	This study does not involve plants
Novel plant genotypes	This study does not involve plants
Authentication	This study does not involve plants

Flow Cytometry

Plots

Confirm that:

☒ The axis labels state the marker and fluorochrome used (e.g. CD4-FITC).

☒ The axis scales are clearly visible. Include numbers along axes only for bottom left plot of group (a 'group' is an analysis of identical markers).

☐ All plots are contour plots with outliers or pseudocolor plots.

☐ A numerical value for number of cells or percentage (with statistics) is provided.

Methodology

Sample preparation	For in vitro experiments: iPSC-derived microglia and THP-1 macrophages were stained in cell culture media using BODIPY (3.7uM, 30 min), LysoTracker (100nM, 30 min), LysoSensor (1uM, 1 min), Live/Dead (1uM, 30 min), DQ-BSA (2uM, 1h) followed by collection. iPSC-derived microglia were collected by gentle pipetting and Accutase treatment (5 min, 37C). THP-1 macrophages were collected using Trypsin-EDTA 0.25% (5 min, 37C). Samples were washed twice in DPBS, and resuspended in FACS buffer (DPBS, 2% BSA fraction V), then filtered through 40um cell strainer and analysed.<br>For in vivo experiments: mice were transcardially perfused with 10ml cold PBS prior to brain collection. Olfactory bulb and cerebellum were discarded, followed by enzymatic digestion of the remaining brain in IMDM medium (Cytiva, SH30259.02) supplemented with 1mg/ml Collagenase type IV (Worthington, LS004186) and 33.3U/ml DNase I (Roche, 11284932001) at 37°C for 45min with occasional mixing and dissociation by pipetting. Subsequently, cells were kept on ice or at 4°C throughout further processing. Enzymatic digestion was followed by filtering through a 70µm cell strainer, washing with icecold<br>2% FCS/PBS and spinning at 300xg for 10min. To remove myelin, the pellet was resuspended in 38% percoll/PBS (Cytiva, GE17-0891-02) and spun at 800xg for 15min (no break). Cells were washed in ice-cold 2% FCS/PBS, followed by FcR block and staining.
Instrument	Attune Flow Cytometer (Thermo Fisher Scientific) (in vitro), BD FACS Aria Fusion (BD Biosciences)
Software	FCS Express 7 Research (De Novo Software by Dotmatics), FACS Diva 4.0 (BD Biosciences)
Cell population abundance	Cell population abundance in post-sorting fraction was not assessed. RNAseq analysis of ascutely isoalted microglia show enrichment of microglial-specific genes over neuron- or astrocyte-specific genes.
Gating strategy	For in vitro experiments: Cells were initially gated on FSC-A and SSC-A, and doublets were excluded. Dead cells were excluded using Live/Dead Fixable Violet Dead Cell Stain Kit. Gates were drown based on fluorescence minus one (FMO) controls for each fluorescent marker (BODIPY, LysoTracker, LysoSensor and DQ.-BSA).<br>For in vivo experiment: Cells were initially gated on FSC-A and SSC-A, and doublets were excluded. Dead cells were excluded using viability dye, gates were drown based on FMO controls. Following gating strategy was used: viability dye-Ly6C-Ly6GMHCII- CD38-CD45.2+CD11b+.

☒ Tick this box to confirm that a figure exemplifying the gating strategy is provided in the Supplementary Information.
